# FNDC5/Irisin Inhibits the Inflammatory Response and Mediates the Aerobic Exercise-Induced Improvement of Liver Injury after Myocardial Infarction

**DOI:** 10.3390/ijms24044159

**Published:** 2023-02-19

**Authors:** Tao Wang, Mengyuan Yu, Hangzhuo Li, Shuguang Qin, Wujing Ren, Yixuan Ma, Wenyan Bo, Yue Xi, Mengxin Cai, Zhenjun Tian

**Affiliations:** 1 Institute of Sports Biology, College of Physical Education, Shaanxi Normal University, Xi’an 710119, China; 2 School of Life Science and Technology, Xi’an Jiaotong University, Xi’an 710049, China

**Keywords:** myocardial infarction, liver injury, inflammation, macrophage, aerobic exercise, irisin

## Abstract

Myocardial infarction (MI) causes peripheral organ injury, in addition to cardiac dysfunction, including in the liver, which is known as cardiac hepatopathy. Aerobic exercise (AE) can effectively improve liver injury, although the mechanism and targets are currently not well established. Irisin, mainly produced by cleavage of the fibronectin type III domain-containing protein 5 (FNDC5), is a responsible for the beneficial effects of exercise training. In this study, we detected the effect of AE on MI-induced liver injury and explored the role of irisin alongside the benefits of AE. Wildtype and *Fndc5* knockout mice were used to establish an MI model and subjected to AE intervention. Primary mouse hepatocytes were treated with lipopolysaccharide (LPS), rhirisin, and a phosphoinositide 3-kinase (PI3K) inhibitor. The results showed that AE significantly promoted M2 polarization of macrophages and improved MI-induced inflammation, upregulated endogenous irisin protein expression and activated the PI3K/ protein kinase B (Akt) signaling pathway in the liver of MI mice, while knockout of *Fndc5* attenuated the beneficial effects of AE. Exogenous rhirisin significantly inhibited the LPS-induced inflammatory response, which was attenuated by the PI3K inhibitor. These results suggest that AE could effectively activate the FNDC5/irisin-PI3K/Akt signaling pathway, promote the polarization of M2 macrophages, and inhibit the inflammatory response of the liver after MI.

## 1. Introduction

Heart failure (HF) is a frequent complication of myocardial infarction (MI), which is one of the leading causes of death worldwide [1]. Clinical studies have found that acute MI leads to impaired liver function, with elevated serum levels of aspartate aminotransferase (AST) in approximately 85.6% of MI patients and alanine aminotransferase (ALT) in 48.2% of MI patients [2,3]. HF could induce the development of acute cardiogenic liver injury and congestive hepatopathy, moreover, the incidence of congestive liver disease in patients with HF post MI is up to 65% [4]. Guzeeva et al. have indicated the abnormalities in the liver of patients with MI, including metabolism disorders and structural changes, might be due to the decreased contractile capacity of the myocardium [5]. There was a significant correlation between the severity of liver injury and mortality after MI [2,3]. Therefore, it is of great significance to improve liver injury in patients with MI.

Inflammation is often a cause of liver injury in many types of liver diseases. In patients with MI, cardiac dysfunction inevitably leads to a decrease in the peripheral blood volume, which would induce cellular ischemia and hypoxia around the hepatic portal vein and an imbalance of redox levels [6,7]. Excessive reactive oxygen species (ROS) trigger an inflammatory response and increase the synthesis and secretion of pro-inflammatory cytokines, such as nuclear factor-kappa B (NF-κB) and tumor necrosis factor-α (TNF-α) [8]. Macrophages are widely distributed innate immune cells, and play a fundamental role in the initiation, maintenance, and resolution of inflammation [9]. It is known that proinflammatory cytokines can promote the polarization of M1-type macrophages, which would further exacerbate the expressions of proinflammatory cytokines and induce the inflammatory response [10]. A previous study showed that MI could trigger systemic inflammation and activate macrophages in the liver, which would be one of the mechanisms of MI-induced liver injury [11]. Studies have revealed that the increased M1 macrophage polarization and reduced M2 macrophage polarization were the important causes of liver injury in multiple liver disease models [12,13,14,15,16]. Therefore, regulating M1/M2 macrophage polarization would be an effective pathway to inhibit inflammation and alleviate MI-induced liver injury.

Exercise training is an effective intervention to improve cardiac function in MI patients [17]. It has been reported that exercise training provides beneficial effects in various liver diseases, including hepatic ischemia-reperfusion injury [18], drug-related liver injury [19], and non-alcoholic fatty liver disease (NAFLD) [20]. The mechanisms were related to inhibiting ROS and inflammation, regulating M1/M2 macrophages polarization, increasing β-oxidation and reducing intrahepatic fat content, hepatocyte autophagy, and apoptosis [18,21,22,23,24]. It has been demonstrated that aerobic exercise (AE) ameliorated liver injury in MI rats by reducing oxidative stress [25]. However, whether exercise training could inhibit the inflammation in the liver after MI needs to be further explored.

Irisin is an exercise-induced myokine, which is cleaved from fibronectin type III domain-containing protein 5 (FNDC5). It has been reported that irisin plays important roles in regulating energy metabolism, insulin resistance, oxidative stress, inflammation, cell apoptosis, and fibrosis [26,27,28]. FNDC5/irisin is involved in different types of liver diseases, such as NAFLD, multiple detrimental insults-induced liver injury, and hepatic malignancy [29]. However, little is known about the effect of irisin on MI-induced liver injury. Exercise training could induce hepatic irisin expression, inhibit the inflammatory response, and improve liver function in NAFLD [30,31]. Whether exercise training could improve MI-induced liver injury through irisin needs to be explored. In this study, we prepared MI models with AE intervention by using wildtype (WT) and *Fndc5* knockout (*Fndc5^-/-^*) mice, and we found that FNDC5/irisin inhibited the inflammatory response and mediated the AE-induced improvement of the liver injury and inflammation after MI.

## 2. Results

### 2.1. AE Inhibited Liver Injury in Mice with MI

**Figure 1:**
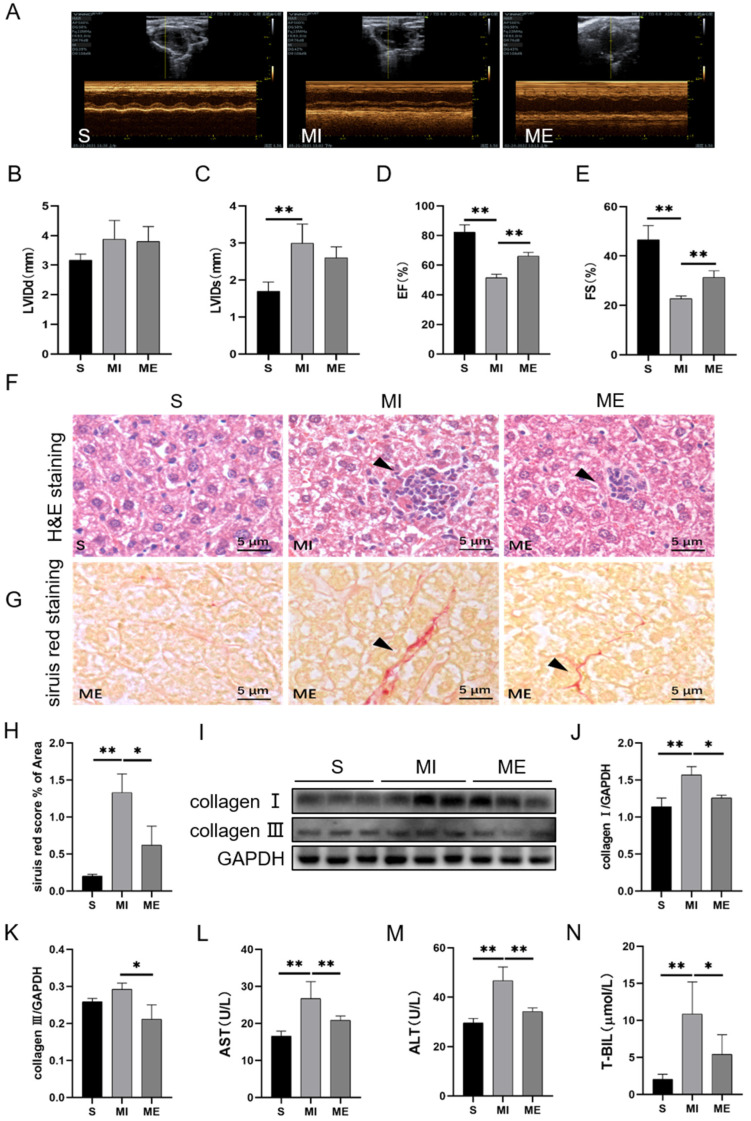
Effects of AE on the structure and function of the liver in mice with MI. (**A**–**E**), results of echocardiography; (**F**), H&E staining of liver tissue, the arrow indicates the area of inflammatory cell infiltration. (**G**,**H**), sirius red staining and analysis of liver tissue and collagen fibers (red), the arrow of Figure (**G**) indicates the area of collagen fiber deposition. (**I**–**K**), the expressions of hepatic collagen I and collagen III. (**L**–**N**), the levels of AST, AST, and T-BIL in serum. Scale bar: 5 μm. Data are expressed as mean ± SEM, n = 6. * *p* < 0.05, ** *p* < 0.01 by one-way ANOVA. S: sham group; MI: myocardial infarction group; ME: MI with AE group.

The WT mice were used to establish the MI model and survival mice were randomly divided into the sham-operated group (S), MI group, and MI with AE group (ME). At first, we evaluated the cardiac function by using echocardiography to confirm the effectiveness of AE on MI mice. The results showed that, compared to the S group, the left ventricular internal diameter at end diastole (LVIDd) had a trend that increased, whereas the left ventricular internal diameter at end systole (LVIDs) increased significantly (*p* < 0.01), ejection fraction (EF) and fractional shortening (FS) decreased significantly (both *p* < 0.01) in the MI group. In comparison to the MI group, AE increased EF (*p* < 0.01) and FS (*p* < 0.01) significantly (Figure 1A–E).

We examined the histological changes in the liver by using H&E staining and sirius red staining. The results showed that, compared to the S group, MI induced inflammatory cell infiltration and increased collagen deposition (*p* < 0.01), and collagen I (*p* < 0.01) expression in the livers of the MI group. Compared to the MI group, AE reduced the degree of inflammatory cell infiltration, decreased collagen deposition (*p* < 0.05), and the expressions of collagen I (*p* < 0.05) and collagen III (*p* < 0.05) in the ME group (Figure 1F–K). We evaluated liver function by detecting serum transaminase levels. Compared to the S group, the levels of AST, ALT, and total bilirubin (T-BIL) in the serums were significantly increased in the MI group (all *p* < 0.01), which were reversed by AE (*p* < 0.01 for AST and ALT, *p* < 0.05 for T-BIL, Figure 1L–N). These results indicated that the MI-induced liver injury and dysfunction were improved by AE.

### 2.2. AE Regulated Macrophage Polarization, Suppressed Inflammatory Response and Activated Irisin/PI3K/Akt Signaling Pathway in the Liver of MI Mice

**Figure 2:**
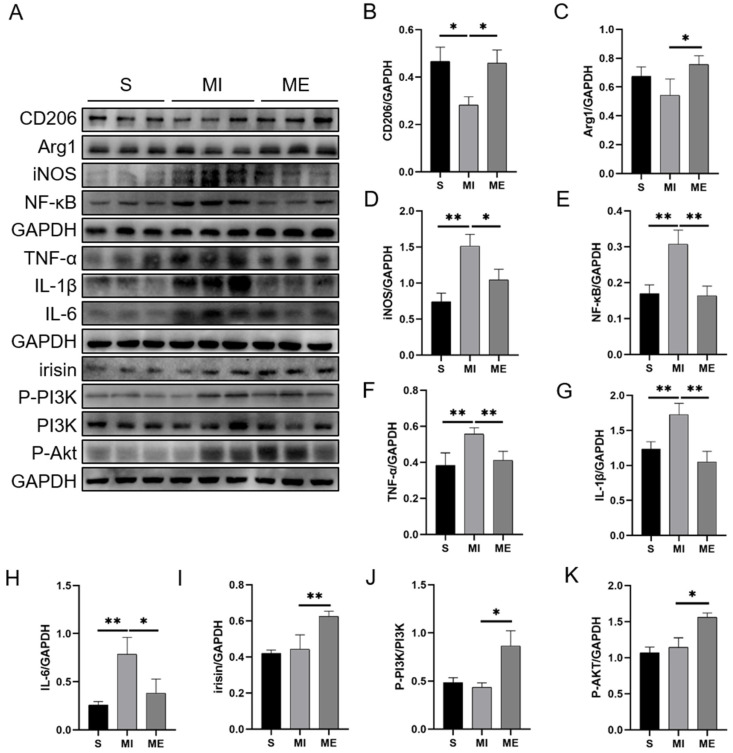
Effects of AE on the macrophage polarization, inflammatory response, and activation of irisin/PI3K/Akt signaling pathway in the livers of the MI mice. (**A**), Western blotting results; (**B**,**C**), analysis of the expressions of CD206 and Arg1. (**D**–**H**): analysis of the expressions of inflammatory factors. (**I**–**K**), analysis of the activation of the irisin–PI3K/Akt signaling pathway. Data are expressed as mean ± SEM, n = 3. * *p* < 0.05, ** *p* < 0.01 by one-way ANOVA. S: sham group; MI: myocardial infarction group; ME: MI with AE group.

To further examine the mechanisms of AE on liver injury, we measured the inflammation level by detecting the expressions of macrophage polarization-related proteins and inflammatory factors in the liver tissues. Mannose receptor (CD206) and arginase-1 (Arg1) were viewed as markers of M2 macrophage [32]. Western blotting results showed that compared to the S group, reduced protein expressions of CD206 (*p* < 0.05) and Arg1 were detected in the MI group, which were upregulated by AE (both *p* < 0.05, Figure 2A–C). Meanwhile, MI increased the expressions of inducible nitric oxide synthase (iNOS, *p* < 0.01), NF-κB (*p* < 0.01), TNF-α (*p* < 0.01), interleukin-1β (IL-1β, *p* < 0.01), and interleukin-6 (IL-6, *p* < 0.01) when compared to the S group. While compared to the MI group, AE significantly reduced the expressions of iNOS (*p* < 0.05), NF-κB (*p* < 0.01), TNF-α (*p* < 0.01), IL-1β (*p* < 0.01), and IL-6 (*p* < 0.05, Figure 2D–H). These results indicated that MI promoted the development of inflammation, while AE promoted M2 macrophage polarization and inhibited inflammation.

To verify whether irisin and the downstream PI3K/Akt signaling pathway were involved in this process, we detected the protein expression of irisin and the phosphorylation of PI3K and Akt. The results showed that AE significantly upregulated the expression of irisin (*p* < 0.01) and the phosphorylation of PI3K and Akt (both *p* < 0.05) in the ME group when compared to the MI group (Figure 2I–K). These results indicated that AE effectively activated the FNDC5/irisin-PI3K/Akt signaling pathway in the livers of the MI mice.

### 2.3. Knockout of Fndc5 Impaired the Protective Effect of AE on MI-Induced Liver Injury

**Figure 3:**
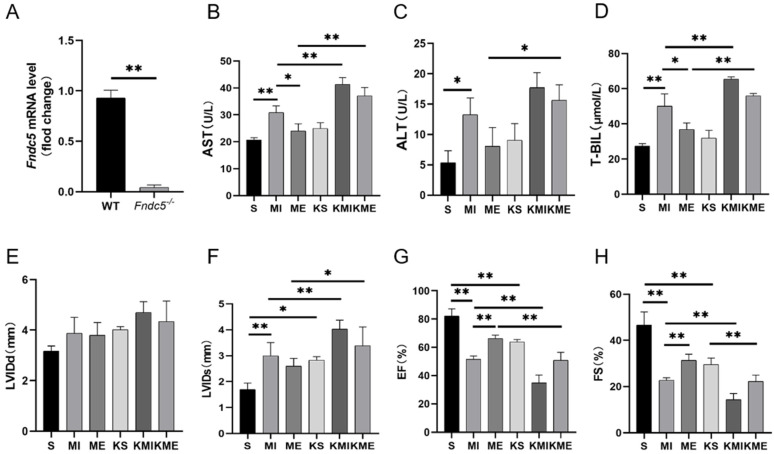
Effect of *Fndc5* knockout on the MI-induced liver injury and the benefits of AE. (**A**), RT-qPCR results of *Fndc5* in the WT and *Fndc5*^-/-^ mice; (**B**–**D**), serum levels of AST, ALT, and T-BIL; (**E**–**H**), hemodynamic results. Data are expressed as mean ± SEM, n = 6. * *p* < 0.05, ** *p* < 0.01 by two-way ANOVA. S: sham group; MI: myocardial infarction group; ME: MI with AE group; KS, KMI, and KME: the S, MI, and ME groups of the *Fndc5*^-/-^ mice.

To determine the exact role of irisin in the protective effect of AE on MI-induced liver injury, we prepared *Fndc5^-/-^* MI mice and intervened them with AE. At first, we detected the expression of *Fndc5* in WT and *Fndc5^-/-^* mice by RT-qPCR. Compared to the WT mice, minimal *Fndc5* was detected in the livers of the *Fndc5^-/-^* mice (*p* < 0.01, Figure 3A). Survival *Fndc5*^-/-^ mice were also divided into the sham group (KS), MI group (KMI), and ME group (KME). Compared with the S, MI, and ME groups of the WT mice, knockout of *Fndc5* mice further increased the levels of AST (both *p* < 0.01 in the KMI and KME groups, Figure 3B), ALT (*p* < 0.05 in the KME group, Figure 3C), and T-BIL (both *p* < 0.01 in the KMI and KME groups, Figure 3D), and reduced cardiac function by increasing LVIDs (both *p* < 0.05 in the KS and KME groups, *p* < 0.01 in the KMI group, Figure 3F) and decreasing EF and FS (all *p* < 0.01 in the KS, KMI and KME groups, Figure 3G,H) in the *Fndc5^-/-^* mice. These results indicated that *Fndc5* knockout aggravated MI-induced liver injury and cardiac dysfunction and inhibited the protective effect of AE, however, it had no significant effect on the liver function of the sham mice. Moreover, the deterioration of cardiac function, due to *Fndc5* knockout, increased the mortality after MI, which resulted in a reduction in sample size.

**Figure 4:**
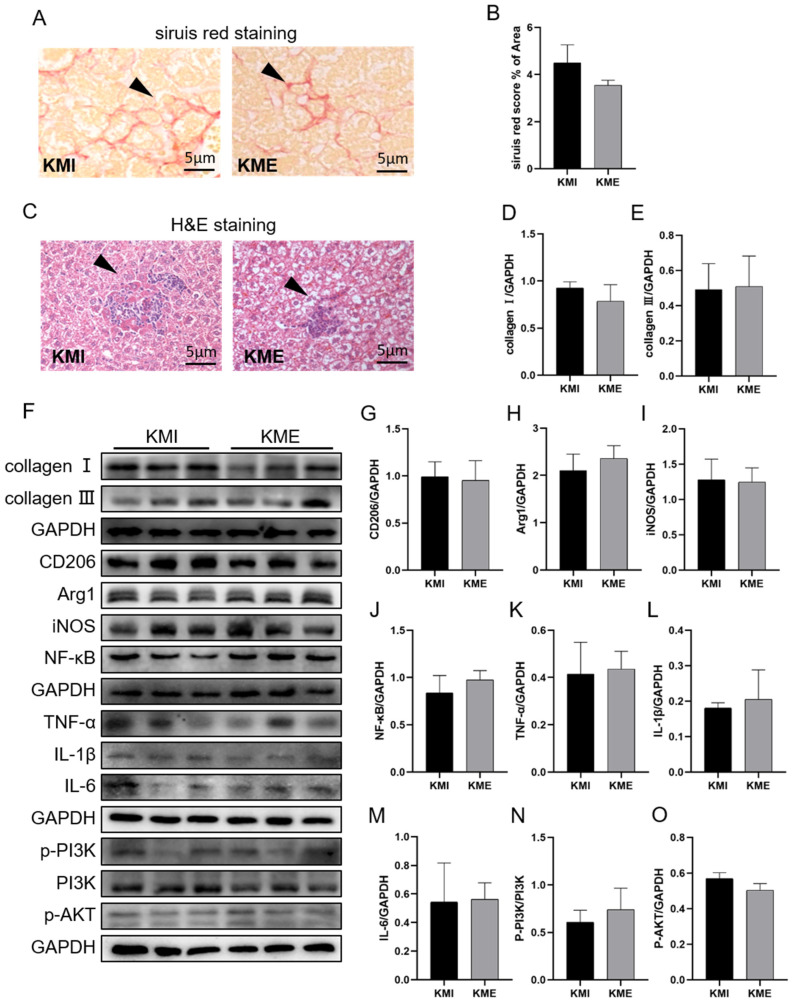
Effect of *Fndc5* knockout on the AE-inhibited inflammatory response in the livers of the MI mice. (**A**,**B**), sirius red staining of liver tissue, the arrow of Figure (**A**) indicates the area of collagen fiber deposition; (**C**), H&E staining of liver tissue, the arrow indicates the area of inflammatory cell infiltration; (**D**,**E**), the expressions of hepatic collagen I and collagen III. (**F**), Western blotting results; (**G**,**H**), analysis of the expressions of CD206 and Arg1. (**I**–**M**): analysis of the expressions of inflammatory factors. (**N**,**O**), analysis of the activation of the PI3K/Akt signaling pathway. Data are expressed as mean ± SEM, n = 3. KMI: myocardial infarction group of the *Fndc5*^-/-^ mice; KME: MI with AE group of the *Fndc5*^-/-^ mice.

Based on these results, we compared the effect of AE on the inflammatory response between the KMI and KME groups. H&E staining, sirius red staining, and western blotting results showed AE has no effect on the levels of inflammatory infiltration, collagen deposition, (Figure 4A–F) and the expressions of CD206, Arg1, NF-κB, iNOS, TNF-α, IL-1β, and IL-6 as well as the phosphorylation of PI3K and Akt (Figure 4F–O). These results showed that the knockout of *Fndc5* attenuated the effects of AE on the inhibition of inflammation and activation of the PI3K/Akt signaling pathway in the livers of the MI mice.

### 2.4. Irisin Activated the PI3K/Akt Signaling Pathway and Inhibited Inflammation In Vitro

**Figure 5:**
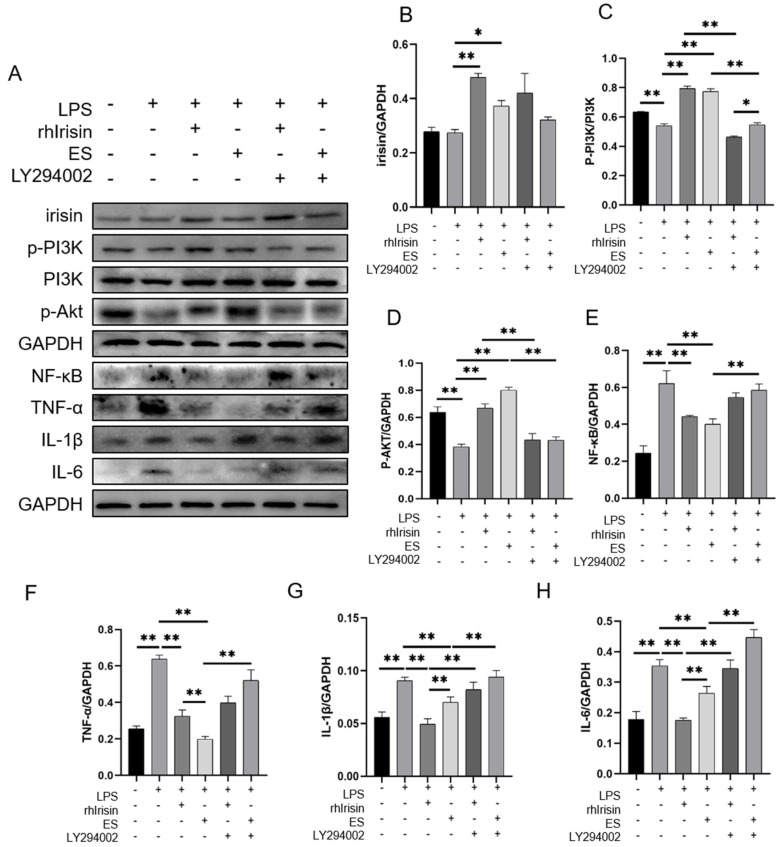
Irisin exerted anti-inflammatory effects through activation of the PI3K/Akt signaling pathway. (**A**), Western blotting results; (**B**–**H**), analysis of the expressions of inflammatory factors and signaling pathway proteins in primary mouse hepatocytes. Data are expressed as mean ± SEM, n = 3. * *p* < 0.05, ** *p* < 0.01 by one-way ANOVA.

To verify whether exogenous irisin could inhibit inflammation, primary mouse hepatocytes were isolated and treated with LPS, rhirisin, exercised serum (ES), and LY294002 (a PI3K inhibitor). The results showed that LPS intervention significantly inhibited the phosphorylation of PI3K and Akt (all *p* < 0.01). Both rhirisin and ES significantly increased the expression of irisin (*p* < 0.01 for rhirisin, *p* < 0.05 for ES), the phosphorylation of PI3K (both *p* < 0.01) and Akt (both *p* < 0.01) in LPS-treated hepatocytes. LY294002 significantly inhibited the phosphorylation of PI3K (both *p* < 0.01) and Akt (both *p* < 0.01) in cells with rhirisin and ES intervention (Figure 5A–D).

LPS intervention significantly increased the expressions of NF-κB (*p* < 0.01), TNF-α (*p* < 0.01), IL-1β (*p* < 0.01), and IL-6 (*p* < 0.01) compared to the control group. Both rhirisin and ES intervention reduced the expressions of NF-κB (both *p* < 0.01), TNF-α (both *p* < 0.01), IL-1β (both *p* < 0.01), and IL-6 (both *p* < 0.01) in the LPS-treated cells (Figure 5A,E–H). The PI3K inhibitor LY294002 inhibited the effects of rhirisin and ES by increasing the expressions of NF-κB (*p* < 0.01 for ES), TNF-α (*p* < 0.01 for ES), IL-1β (both *p* < 0.01), and IL-6 (both *p* < 0.01, Figure 5A,E–H). In addition, the results showed that rhirisin and ES could activate the PI3K/Akt signaling pathway and inhibit the LPS-induced inflammatory response in primary mouse hepatocytes.

## 3. Discussion

MI-induced liver injury has been confirmed in clinical patients and animal models. Exercise training can improve MI-induced liver injury, although the mechanisms have not yet been fully elucidated. In this study, we focused on the role of irisin in the anti-inflammatory effect of AE in the livers of MI mice. The main findings of this study were as follows: (1) AE inhibited MI-induced inflammation and dysfunction, upregulated irisin protein expression, and activated the PI3K/Akt signaling pathway in the livers of MI mice; (2) knockout of *Fndc5* alleviated the benefits of AE in MI-induced liver injury and the inflammatory response; (3) rhirisin improved LPS-induced inflammation through the PI3K/Akt signaling pathway in hepatocytes. These results revealed the anti-inflammatory effect of irisin in liver injury after MI and provide a basis for screening the exercise targets in the protective effect on MI-induced liver injury.

Left ventricular systolic dysfunction after MI causes cardiac insufficiency effects. The ensuing ischemia and hypoxia both reduce cardiac function and induce damage to the other organs, such as the kidney, brain, skeletal muscle, and liver [2,33,34]. It has been shown that considerable changes were observed in the rat liver six months after MI [35,36]. Acute and chronic HF may lead to acute ischemic hepatitis or chronic congestive hepatopathy. In this study, we found inflammatory cell infiltration and collagen deposition in the liver tissue during the seventh week of MI. Meanwhile, serum transaminase levels increased significantly. These results confirmed MI could induce liver injury. Six weeks of AE inhibited liver injury, which would block the further development of the injury and liver tissue remodeling.

Hypoxia usually increases the production of ROS, leading to oxidative damage, which further promotes inflammation. Overproduction of ROS in the liver induces macrophage polarization to the M1 type. M1 macrophages are involved in proinflammatory responses by producing proinflammatory cytokines (IL-1β, IL-6, IL-12, IL-18, and TNF-α) and chemokines to guide acute inflammatory responses, in contrast, M2 macrophages play an anti-inflammatory role [37,38]. One study demonstrated that AE could reduce oxidative stress in the liver of MI rats and ameliorate liver injury [25]. However, little is known about the effect of AE on macrophage polarization and the inflammatory response in the liver after MI. In this study, our results confirmed that MI reduced the increase of M2 macrophages, and upregulated the expressions of iNOS, NF-κB, TNF-α, IL-1β, and IL-6. AE significantly increased M2 macrophages and inhibited the expressions of inflammatory factors. This all suggests that AE is an effective method to inhibit inflammation in the liver after MI by regulating macrophage polarization.

Many studies focused on the crosstalk between the liver and heart [4,39,40] and found liver-derived cytokines, such as fibroblast growth factor 21, IL-22, proprotein convertase subtilisin/kexin type 9, and coagulation factor XI participated in cardiac protection after MI [41,42,43]. However, few studies indicated the effect of cytokines on MI-induced liver injury. Irisin is a well-studied myokine, which was discovered in 2012 [26]. Irisin is mainly released by skeletal muscle and adipose tissue and is also expressed in the heart, liver, spleen, pancreas, brain, and kidney [44]. Studies have suggested the therapeutic potential of irisin against a variety of liver diseases based on its antioxidative, antiapoptotic, and anti-inflammatory functions [45]. Exogenous irisin treatment was effective in protecting the liver from ischemia-reperfusion and sepsis-induced injury [46,47]. Moreover, irisin could alleviate LPS-induced liver injury and inflammation by inhibiting NLR family pyrin domain containing 3 (NLRP3) and NF-κB signaling [48]. It has been reported that exercise-induced irisin inhibited inflammation and improved liver injury in NAFLD [31]. In this study, we found less expression of irisin in the livers of the mice with MI, which could be upregulated by AE. Moreover, knockout of *Fndc5* attenuated AE-inhibited inflammation and liver injury after MI. Our data suggest that endogenous irisin played an irreplaceable role in the improvement of MI-induced liver injury by AE.

The PI3K/Akt signaling pathway is a classical intracellular signaling pathway that plays a crucial role in the survival, proliferation, migration, and polarization of macrophages [49,50,51]. In addition, PI3K/Akt is important to inhibit liver fibrosis, inflammation, oxidative stress, and apoptosis, as well as in promoting hepatocyte regeneration [52,53,54,55]. Irisin could regulate hepatic glucose metabolism via PI3K/Akt activation [56], suggesting that the PI3K/Akt signaling pathway is an important downstream signal of irisin. Previous studies found that the activation of the PI3K/Akt signaling pathway during liver ischemia-reperfusion injury, increased IL-4, and IL-10 expressions, decreased IL-1β and TNF-α expressions, and reduced the hepatic inflammatory response. In contrast, inhibition of PI3K/Akt signaling increased NF-κB transcription and the release of TNF-α, IL-1β, and IL-6, ultimately aggravating liver injury [57,58]. Similar to these studies, our animal experiments suggested that AE upregulated irisin expression and inhibition of the inflammatory response were related to the activation of the PI3K/Akt signaling pathway. Furthermore, inhibition of the PI3K/Akt signaling pathway attenuated the anti-inflammatory effect of irisin in vitro. However, we have no direct evidence to show how irisin inhibited inflammation by activating the PI3K/Akt signaling pathway.

Studies have shown that the PI3K/Akt signaling pathway can inactivate Toll-like receptor 4 (TLR4) by preventing the recruitment of the Toll-IL-1 resistant structural domain attachment protein (TIRAP) to the cell membrane, which would inhibit the activation of NF-κB and its downstream proinflammatory cytokines [59,60,61]. Moreover, it has been shown that the anti-inflammatory effect of irisin was connected with the TLR4/myeloid differentiation factor 88 (MyD88) signaling pathway [62]. Based on these, TLR4 would play an important role in the inhibition of the inflammation of the irisin-PI3K/Akt signaling. Actually, it still has a different view, whereby irisin inhibited the PI3K/Akt/NF-κB signaling pathway to ameliorate inflammation, such as in chondrocytes [63]. We believed that the functions of irisin and the PI3K/Akt signaling pathway were related to the types of tissue and pathological microenvironment of diseases. The exact mechanisms of the irisin-mediated anti-inflammatory effect of AE, in the livers of MI mice, still require more in-depth studies.

## 4. Materials and Methods

### 4.1. Animals and Exercise Protocol

Eight-week-old male C57BL/6J wildtype (WT) mice were purchased from the laboratory animal center of the Xi’an Jiaotong University (Xi’an, China). The *Fndc5^+/-^* mice (C57BL/6N-*Fndc5^em1Cya^*, S-KO-09897), which were conventional knockout by CRISPR-Cas9, were purchased from Cyagen Biosciences Inc. (Guangzhou, China) and were used to generate the homozygous target mice. The sequence of primers for screening homozygous mice is as follows: F1: 5′-CTGTCTCCAATGTTCCACT TGTCTG-3′; R1: 5′-CTTGCCTTTGTTCTTTGAGGCCATC-3′; R2: 5′-GCTTGAACCAAGGCGAGAGCTAGT-3′. All animals were housed in the Institute of Sports Biology, Shaanxi Normal University (temperature: 23–25 °C and humidity: 40–60%), with four to five animals per cage, who resided under a 12 h light/12 h dark cycle and received ad libitum access to water and standard rodent chow. All experimental protocols were approved by the Ethics Committee of Shaanxi Normal University.

WT and *Fndc5^-/-^* mice were used to establish the MI model by ligation of the left anterior descending coronary artery at the position approximately 2 mm under the junction of the pulmonary conus and left atrial appendage. Surviving WT mice were randomly divided into the sham-operated group (S), MI group, and MI with AE group (ME), n = 6; the surviving *Fndc5^-/^*^-^ mice were also divided into the S group (KS), MI group (KMI), and ME group (KME), n = 3.

Mice in ME and KME groups were subjected to six weeks of treadmill AE from the second week after surgery. The exercise training protocol was based on a previous study [64] and adjusted according to the state of the exercised mice. During the first five days, mice were subjected to adaptive training, in which the speed and duration were gradually increased from 5 m/min for 10 min to 10 m/min for 50 min. The formal training speed was 10 m/min for 60 min per day, five days per week, for six weeks, corresponding to a moderate intensity exercise, and the maximum oxygen uptake was about 65–70% [65,66]. No mice died during the process.

### 4.2. Echocardiographic Measurements

Echocardiography was used to test cardiac function on the second day after ligation and the second day after the last training. Mice were placed in the supine position and anesthetized with isoflurane (3% induction and 1% maintenance, 1 L/min oxygen). The left ventricle internal dimension diastole (LVIDd), the left ventricle internal dimension systole (LVIDs), and the ejection fraction (EF) were recorded by averaging six consecutive cardiac cycles with an ultrasound probe after hair removal. Fractional shortening (FS) was calculated by the formula: FS = (LVIDd − LVIDs)/LVIDd.

### 4.3. Histological Staining and Analysis

Mice were sacrificed and the livers were quickly collected, cleaned with phosphate buffer saline (PBS, pH = 7.2), and fixed in cold 4% formaldehyde or liquid nitrogen for subsequent experiments. Liver tissues fixed in 4% paraformaldehyde were paraffin-embedded. Embedded tissues were subjected to 5 µm serially sectioned and stained with H&E or sirius red, according to the standard procedures.

### 4.4. Primary Mouse Hepatocyte Isolation and Cell Culture

The method of primary mouse hepatocyte isolation was modified from the classic two-step collagenase perfusion technique [67]. The specific steps were as follows: culture plates were covered with 0.01% rat-tail collagen (C8062, Solarbio, Beijing, China) one day before cell isolation. Before separating the cells, the pH of the Perfusion Solution I (0.019 g EGTA dissolved in 100 mL D-HANKS) and Perfusion Solution II (type IV collagenase 40 mg dissolved in 100 mL high sugar DMEM) were adjusted to 7.2–7.4 and placed in a water bath at 40 °C for 1 h. Perfusion Solution I and II were infused retrograde through the inferior vena cava using a 4.5-gauge infusion needle to the liver, which was showing an earthy color and collapsing. The liver was cut out and the gallbladder was removed. The liver tissue was transferred to a culture plate, the liver envelope torn, and gently shaken until the hepatocytes were separated. Hepatocyte suspension was obtained by filtration using a 70 μm cell sieve and centrifuged twice at 500 r/min. After adjusting the density, 1 × 10^6^ hepatocytes were inoculated into each plate and incubated at 37 °C for 8 h. When the cell density reached 80–90%, the primary mouse hepatocytes were treated with LPS (500 ng/mL, 12 h), rhirisin (250 ng/mL, 24 h), exercise serum from mice (1%, 24 h, mimic the effects of AE), and LY294002 (10 μM, 24 h).

### 4.5. Measurement of Liver Function

The ATS (C010-2-1, NanJing JianCheng Bioengineering Institute, Nanjing, China), ALT (C009-2-1), and T-BIL (C019-1) assay kits were used for testing the liver function. The operation procedure was carried out in strict accordance with the instructions.

### 4.6. RT-qPCR

Total RNA was extracted from the frozen liver tissues (15–20 mg) with the RNAeasy^TM^ animal RNA isolation kit with spin columns (R0024FT, Beyotime, Shanghai, China). A RevertAid first-strand cDNA synthesis kit (K1622, Thermo Scientific, Waltham, MA, USA) was used to transcribe mRNA into cDNA. RT-qPCR was performed by using SYBR Green PCR Master Mix (Beyotime) and a CFX96 Real-Time PCR System (Bio-Rad, Hercules, CA, USA). The primers were synthesized by Sangon (Shanghai, China). The primer sequences were as follows: *Fndc5* (F:5′-GGCTGGGAGTTCATGTGGAA-3′; R:5′-TGGGAAGCGGTTATCTTTGCT-3′), *Gapdh* (F:5′-CAGTGCCAGCCTCGTCTCAT-3′; R:5′-AGGGGCATCCACAGTCTTC-3′).

### 4.7. Western Blotting

Extracted tissue protein and cellular protein were separated by 10–12% SDS-PAGE at a constant voltage of 90 V for 90 min, followed by electrotransfer (300 mA, 4 °C, 100 min) to NC membranes (Millipore, Bedford, MA, USA). Membranes were blocked with 5% skim milk for 90 min at room temperature (RT), then, incubated with primary antibodies at 4 °C overnight. The primary antibodies and concentrations were as follows: irisin (1:1000, ab174833, Abcam, Cambridge, UK) NF-κB p65 (1:1000, 10745-1-AP, Proteintech, Rosemont, IL, USA), TNF-α (1:500, 60291-1-Ig, Proteintech), IL-6 (1:1000, 21865-1-AP, Proteintech), IL-1β (1:1000, 16806-1-AP, Proteintech), phospho-PI3K (1:1000, AP0854, ABclonal, Wuhan, China), PI3K (1:1000, ab278545, Abcam), phospho-Akt (1:1000, 28731-1-AP, Proteintech), CD206 (1:1000, 18704-1-AP, Proteintech), Arg-1 (1:1000, 66129-1-Ig, Proteintech), iNOS (1:1000, 22226-1-AP, Proteintech), collagen I (1:1000, 14695-1-AP, Proteintech), and collagen III (1:1000, 22734-1-AP, Proteintech). GAPDH was used as a loading control for protein normalization. On the second day, the membranes were washed three times with Tris-buffered saline with Tween 20 (TBST) and, then, incubated with the HRP-conjugated secondary antibody for 90 min at RT. After washing the membranes, the reactive bands were detected using enhanced chemiluminescence reagent (ECL, Bio-Rad), and visualized using a digitalized Bio-Rad ChemiDocTM MP Imaging system (Bio-Rad).

### 4.8. Statistical Analysis

Image J software was used to process and analyze the microscope images and detect the grayscale values of the bands. GraphPad Prism 5.0.1 was used to analyze experimental data, including T-test and one-way ANOVA followed by Tukey’s test or two-way ANOVA. Data were expressed as the mean ± standard error (SEM) with statistically significant differences selected at the *p* < 0.05 and *p* < 0.01 levels.

## 5. Conclusions

**Figure 6:**
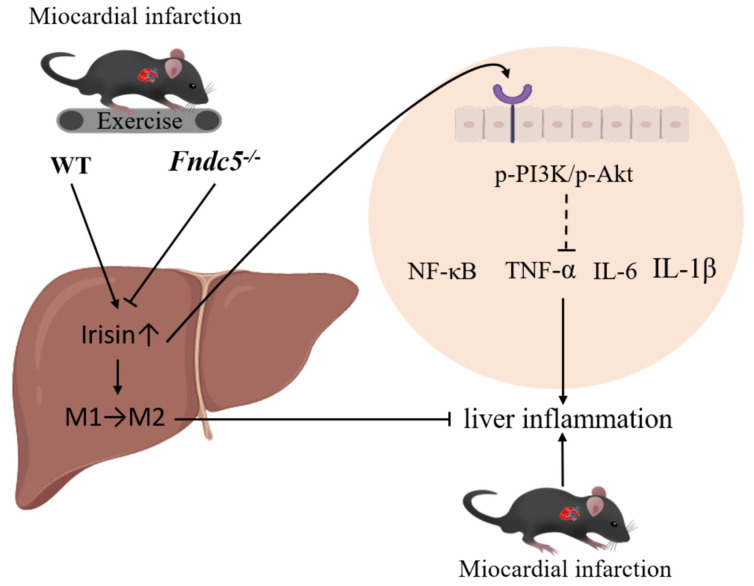
AE ameliorates MI-induced liver inflammatory injury.

In this study, we found AE activated the expression of FNDC5/irisin, activated the PI3K/Akt signaling pathway, promoted the polarization of the M2 macrophages, and inhibited the inflammatory response of the liver after MI. The specific intracellular molecular mechanism needs to be further explored (Figure 6).

## Data Availability

Due to the nature of this research, participants of this study did not agree for their data to be shared publicly, so supporting data is not available.
